# Development of Reduced‐Sodium Cooked Ham Using NaCl Substitutes: Impact on Instrumental and Sensory Properties

**DOI:** 10.1111/1750-3841.71358

**Published:** 2026-08-15

**Authors:** Simoni Cristina Mendes Cardim, Gilmar Freire da Costa, Marise Aparecida Rodrigues Pollonio, Jorge Herman Behrens, Juliano Lemos Bicas

**Affiliations:** ^1^ School of Food Engineering Universidade Estadual de Campinas Campinas São Paulo Brazil

**Keywords:** acceptance, cooked ham, flavor, sodium reduction, texture, transglutaminase

## Abstract

This study aimed to develop cooked ham with less than 600 mg sodium/100 g through partial replacement of NaCl by combinations of potassium chloride (KCl), commercial meat seasoning, and transglutaminase. Eight formulations were produced and evaluated for physicochemical, technological, microbiological, and sensory characteristics. Sodium content was reduced by approximately 30%, decreasing from 863 mg/100 g in the standard formulation to 581–734 mg/100 g in the reformulated products. The reduced‐sodium control exhibited higher cooking loss (8.8%) than the standard formulation (5.2%), whereas formulations containing meat seasoning and transglutaminase reduced cooking loss to values as low as 3.7%. Texture profile analysis showed that transglutaminase increased product firmness, with chewiness increasing from 177.6 N in the reduced‐sodium control to 590.5 N in formulation F5. In relation to sensory evaluation, the standard formulation showed the highest acceptance, followed by formulations F6 and F5, although all reformulated products achieved mean acceptance scores above 6.0 on the 9‐point hedonic scale. Formulations containing 0.35% KCl, 0.25% meat seasoning, and transglutaminase provided the best balance between technological performance and consumer acceptance. Despite the technological improvements, lower saltiness, rubbery texture, and paler pink color remained the main sensory limitations of the reduced‐sodium products. This could be further improved in future trials by fine‐tuning commercial meat flavoring and transglutaminase levels.

**Practical Applications:**

This study shows that the meat industry can produce cooked ham with reduced sodium content using salt substitutes, resulting in healthier and technologically viable products. For consumers, this may provide lower‐sodium alternatives with potential health benefits, if formulation adjustments maintain or improve flavor, texture, and color.

## 1 Introduction

Cooked ham is the third most consumed charcuterie in Brazil, while in Europe, it accounts for 26% of the volume of meat products sold (Los et al. 2014; Arenas et al. 2023). Product quality depends on several factors, among which sodium chloride (NaCl) plays a key role. In processed meats, NaCl inhibits microbial growth, reduces water activity, facilitates protein solubilization, and enhances flavor (Fraqueza et al. 2024). However, excessive sodium intake is associated with chronic diseases such as hypertension, cardiovascular diseases, kidney failure, and osteoporosis (Halim et al. 2020). It is estimated that 2.5 million deaths could be prevented annually through reductions in global salt consumption (WHO 2020). Consequently, guidelines and regulations have encouraged sodium reduction in processed foods. Nevertheless, reducing NaCl remains a technological challenge because it can impair myofibrillar protein extraction, protein binding, yield, texture, and color (Guo et al. 2024; Xiang et al. 2025).

Ruusunen et al. (2001) reported that cooked ham formulated with 1.1% added NaCl exhibited greater cooking losses and significantly lower perceived saltiness than ham containing 1.7% NaCl, indicating that concentrations close to 1.0% approach the lower technological and sensory limit for this type of product. Consistently, Barbieri et al. (2016) demonstrated that cooked ham containing 1.0% NaCl could still achieve acceptable flavor and sensory characteristics when the formulation and cooking process were appropriately optimized, despite some reduction in processing yield and increased dryness. Taken together, these findings support the use of 1.0% NaCl as a practically acceptable minimum level for cooked ham, below which technological performance and sensory quality may be substantially compromised.

Several strategies have been proposed to compensate for the negative effects of NaCl reduction in meat products. Partial replacement with KCl is among the most common approaches and has been shown to improve water binding and yield while maintaining acceptable sensory quality, even when up to half of the NaCl is replaced (Inguglia et al. 2017). Other salts, such as CaCl_2_ and MgCl_2_, may also be used, although they can impart bitter, metallic, or astringent flavors (Şimşek et al. 2023). Calcium salts have additionally been associated with improvements in tenderness and water retention capacity, although some combinations may accelerate lipid and myoglobin oxidation (Gong et al. 2023; Wang et al. 2023). To minimize sensory drawbacks, flavor enhancers and metallic flavor maskers are frequently incorporated, with only a minor contribution to total sodium content (Jia et al. 2024).

Among flavor enhancers, monosodium glutamate (MSG), nucleotides, amino acids, peptides, and protein hydrolysates are commonly used. MSG is particularly effective in sodium‐reduction strategies because it enhances flavor perception and consumer acceptability while allowing substantial sodium reductions (Couto Rosa et al. 2021). Likewise, flavoring mixtures containing glycine and yeast extract have been successfully applied to sodium‐reduced hams, minimizing changes in texture and appearance, although yeast extract may cause undesirable color changes (Delgado‐Pando et al. 2019).

Another strategy involves the use of transglutaminase, which promotes protein cross‐linking, improving gel strength, cohesion, texture, and water retention in reduced‐sodium meat products (Zhu et al. 2025). Previous studies demonstrated that transglutaminase prevented syneresis and improved water retention in dry‐cured ham (Fulladosa et al. 2009; Jira et al. 2017). In cooked ham, NaCl reductions of up to 30% combined with 0.1% transglutaminase maintained water retention capacity, exudation losses, syneresis, and color while improving texture attributes such as hardness and chewiness (Silva 2020). Although several approaches have been proposed to produce sodium‐reduced ham, few studies have combined these strategies. Therefore, this study aimed to produce cooked ham with reduced sodium content (less than 600 mg sodium/100 g) while preserving sensory acceptability and technological quality through the combined use of transglutaminase, meat seasoning, and KCl.

## 2 Material and Methods

### 2.1 Cooked Ham Production

The study was conducted in a food product development laboratory equipped with small‐scale processing equipment for prototype production. The laboratory is located at the headquarters of a company based in São Paulo, São Paulo State, Brazil, and belongs to an organization specializing in the development and commercialization of ingredients for the food industry, including seasonings, processing aids, and enzymes. Due to operational constraints and the objective of evaluating the formulations under real industrial conditions, a single production batch was manufactured for each treatment. The cooked ham formulation used in this study was based on industrial formulations commonly employed in restructured meat products, especially reduced‐sodium products, aiming to maintain technological stability, texture, water‐holding capacity, and sensory acceptability. Extra clean pork meat was used as the main raw material (62.5%), while water/ice (31.475%) was added to facilitate protein extraction, improve emulsion stability, and assist temperature control during processing. Sodium chloride was added at 1.0%, characterizing the formulation as a reduced‐sodium product. This concentration was selected based on previous studies (Barbieri et al. 2016), given that NaCl is essential for the extraction of salt‐soluble myofibrillar proteins, contributing to water‐holding capacity, binding capacity, cooking yield, texture, and sensory quality. Soy protein isolate and carrageenan were incorporated to improve water retention, emulsion stability, texture, and sliceability. Tripolyphosphate and sodium polyphosphate were used to enhance protein functionality and reduce cooking losses. MSG was used as a flavor enhancer to compensate for reduced salt content. The commercial ham seasoning (California 350 ham seasoning) consisted of a blend of spices and sodium‐based curing compounds (salt [91.60%], sugar [5.10%], MSG as a flavor enhancer [0.80%], garlic powder [1.20%], onion powder [0.80%], clove oleoresin [0.30%], and cinnamon essential oil [0.20%], used to improve flavor and sensory characteristics). Hungarian Powder III (Adeste) contained refined salt (93%–95%), sodium nitrite (5%), and sodium nitrate (1%). Both ingredients are commercially available blends commonly used in the manufacture of cooked, cured meat products. Cochineal carmine dye was added to standardize product color. The cooked ham production steps were adapted from Pardi et al. (1996), using the ingredients described in Table 1. Each treatment was produced individually in 5‐kg batches and further divided into four individual hams per treatment, one intended for microbiological analyses and three for the remaining analyses. The products were individually packaged in Cryovac cook‐in bags, followed by molding, pressing to improve product shape, and cooking. The brine was prepared separately for each treatment, and the ingredients were homogenized using a mixer. Phosphates were added first to facilitate protein extraction and improve functional properties, followed by the incorporation of the remaining ingredients. Antioxidants, curing salts, and, when applicable, ACTIVA BFB/BFB* and SALT ANSWER M were added last to ensure proper distribution and stability within the meat matrix.

**TABLE 1:** Standard formulation of cooked ham and brine, and calculation of sodium content in the final product.

Ingredient	Percentage	Brine	mg sodium/100 g of cooked ham
	%	%	
Extra clean pork ham	62.5	0	63.68
Soy protein isolate	2.00	5.33	n.s.
Salt (NaCl)	1.00	2.67	390.0
California 350 ham seasoning	0.65	1.73	222.95
Sucrose	0.70	1.87	n.s.
Monosodium glutamate	0.30	0.80	36.465
Carrageenan	0.50	1.33	n.s.
Tripolyphosphate and sodium polyphosphate	0.50	1.33	85.00
Sodium erythorbate	0.20	0.53	6.70
Hungarian Powder III	0.15	0.40	54.41
Cochineal carmine dye	0.025	0.067	n.s.
Water/ice	31.475	83.93	n.s.
Total	100	100	859.2

*Note*: n.s. = nonsignificant contribution (<1 mg/100 g). California 350 ham seasoning consisted of spices and sodium‐based curing compounds. Hungarian Powder III consisted of sodium chloride and sodium nitrite.

After trimming, 10% of the total meat mass was ground through a 3‐mm plate to simulate fine meat cuts and enhance protein extraction, while the remaining portion was ground through a 14‐mm plate to obtain larger particles, improving brine distribution and product texture. The use of different particle sizes is a common strategy in restructured meat products and cooked ham manufacturing to optimize binding properties and water retention (R. Zhang et al. 2024). The meat batter was weighed and transferred to a rotary tumbler together with the brine solution and mixed at 26 rpm for 10 h under vacuum (6 bar) and refrigeration (4°C–6°C), conditions that favor salt diffusion, protein solubilization, and improved yield (Ge et al. 2022). Cooking was performed in a water tank (Phase 1: 65°C for 30 min; Phase 2: 72°C for 90 min; Phase 3: until the internal temperature reached 72°C), according to conventional cooked ham processing conditions described for cured meat products (R. Zhang et al. 2024). Subsequently, the cooked ham was cooled and stored under refrigeration (5°C–8°C) for 24 h before unmolding, portioning, and vacuum packaging for analysis. At least three pieces were prepared for each formulation.

For the ham formulations with reduced sodium content, NaCl in the standard formulation (Table 1) was partially replaced with other components for the test formulations, as presented in Table 2.

**TABLE 2:** Content of key components (NaCl, transglutaminase Activa, Salt Answer meat condiment, and KCl) in the different formulations of cooked ham. The proportion of the other components was the same as presented in Table S1.

Formulation	NaCl (%)	ACTIVA BFB* (%)	ACTIVA BFB (%)	SALT ANSWER M (%)	KCl (%)	Calculated sodium content (mg/100 g)
Standard (S)	1.0000	—	—	—	—	859.2
Reduced‐sodium control (C)	0.2979	—	—	—	—	585.4 (sodium reduction of 31.9%)
Formulation 1 (F1)	0.2979	0.0450	—	—	—	
Formulation 2 (F2)	0.2979	—	0.0450	—	—	
Formulation 3 (F3)	0.2412	—	—	1.0000	—	
Formulation 4 (F4)	0.2411	—	0.0450	1.0000	—	
Formulation 5 (F5)	0.2837	—	0.0450	0.2500	0.3500	
Formulation 6 (F6)	0.2837	—	0.0225	0.2500	0.3500	

### 2.2 Proximate Composition and Physicochemical Analyses of Cooked Hams

Carbohydrates, proteins, fats, fixed mineral residue (ash), moisture, and volatiles were analyzed according to the method described in Zenebon et al. (2008). Sodium determination followed the AOAC 2011.14 method based on coupled plasma optical emission spectrometry (ICP‐OES) (AOAC International 2011). Water activity (*a*_w_), pH, and the moisture/protein ratio were calculated in accordance with the recommendations of the Manual of Official Methods for Analysis of Foods of Animal Origin (MAPA 2022).

### 2.3 Quality and Processing Analyses

#### 2.3.1 Cooking Loss

Cooking loss was determined by weighing the samples before and after cooking using a semi‐analytical balance. Measurements were performed in triplicate for each formulation, and cooking loss was calculated according to Equation (1):

(1) $$\mathrm{CL}\;\left(\%\right)=\frac{W_{i}-W_{f}}{W_{i}}\;\times 100,$$

where CL (%) is the cooking loss, *W*_i_ is the initial ham weight, and *W*_f_ is the final (after cooking) weight.

#### 2.3.2 Sliceability

Sliceability was evaluated using a slicer (Hobart) and a semi‐analytical balance, according to a procedure adapted from Prestes (2008). Forty slices, each 1 mm thick, were obtained from each formulation. The slices and the remaining piece were weighed, and the weight difference was considered the loss generated during slicing. The result was expressed as the percentage of mass loss per formulation, according to Equation (2):

(2) $$L_{s}\;\left(\%\right)=\frac{W_{\mathrm{bs}}-W_{\mathrm{as}}}{W_{\mathrm{bs}}}\times 100,$$

where *L*_s_ (%) is the percentage of mass loss resulting from slicing, *W*_bs_ is the weight of the cooked ham before slicing, and *W*_as_ is the combined weight of the slices and the remaining piece after slicing.

#### 2.3.3 Expressible Water (EW)

This procedure was adapted from the methodology developed by Lucherk et al. (2017), in which a cube with an edge of 1 cm is removed from the central part of the sample and pressed against two qualitative filter papers. The samples were prepared one by one, and the filter paper (Unifil 18.5 cm; Unifil ETQ) was previously dried (105°C/24 h) and cooled in a desiccator. Initially, the paper was weighed, and the prepared sample was added; then both were compressed for 30s at 78.45N using a universal texturometer, model TA.XT “Plus Texture Analyzer.” After compression, the sample was removed from the filter paper, and the paper containing the exudate was weighed again. All weighing was carried out on an analytical balance. The percentage of fluid weight lost from the sample during compression was quantified as the sample juiciness measurement and expressed as % expressible water of treatments according to Equation (3):

(3) $$\mathrm{Expressible}\;\mathrm{water}\;\left(\%\right)=\frac{\mathrm{PF}W_{i}-\mathrm{PF}W_{f}}{\mathrm{PF}W_{i}}\times 100,$$

where PFW_i_ is the initial weight of the filter paper, and PFW_f_ is the final (after pressing) weight of the filter paper.

Three pieces of cooked ham were taken from each formulation, and five samples were taken from each piece, totaling 15 analytical responses per formulation at each time point (2, 15, and 30 days after manufacture).

#### 2.3.4 Objective Color

Color was measured using a HunterLab Colorflex EZ spectrophotometer based on the CIELAB color system established by the Commission Internationale de L'éclairage (CIE). Measurements were performed using illuminant D65, a 2° observer angle, RSEX mode, and an aperture diameter of 10 mm (0.4 inch). The CIELAB parameters L* (lightness), a* (red/green coordinate), and b* (yellow/blue coordinate) were measured in triplicate. Three slices, approximately 5 mm thick, were removed from the central portion of each cooked ham. The total color difference (ΔE*_ab_) was calculated using the standard formulation as the reference, according to Equation (4) (Silva 2020):

(4) $$\Delta =\Delta E_{ab}^{*}={\left[{\left(L_{P}^{*}-L_{a}^{*}\right)}^{2}+{\left(a_{P}^{*}-a_{a}^{*}\right)}^{2}+{\left(b_{P}^{*}-b_{a}^{*}\right)}^{2}\right]}^{1/2},$$

where L*_P_, a*_P_, and b*_P_ and L*_a_, a*_a_, and b*_a_ are the values of L*, a*, and b* of the standard formulation and the sample, respectively.

#### 2.3.5 Texture Analysis

Texture profile analysis (TPA) was performed using a TA.XT Plus Texture Analyzer (Stable Micro Systems, Godalming, UK). Cylindrical samples measuring 2 cm in diameter and 2 cm in height were compressed twice along their length to 50% of their original height using a 3.7‐cm‐diameter cylindrical aluminum probe. The compression speed was 180 mm/min (3 mm/s). The following parameters were obtained using the instrument software: hardness, springiness, cohesiveness, gumminess, chewiness, and resilience (Romero de Ávila et al. 2014; Ramos and Gomide 2007). Three repetitions were performed for each of three replicates per formulation, totaling nine responses per formulation.

### 2.4 Microbiological Analyses

Microbiological analyses were performed in an external laboratory prior to the sensory evaluation of each formulation, in accordance with the recommendations of Resolution RDC no. 331, dated December 23, 2019. The methodology used to analyze each microorganism was as follows: BAM Ch 4 (adapted) for *Escherichia coli* (Feng et al. 2002); ISO 6888‐1:2021 for Coagulase Positive *Staphylococcus* (ISO 2021); ISO 7937:2004 for *Clostridium perfringens* (ISO 2004); AOAC 2011.03 for *Salmonella* spp.; ISO 21527‐2:2008 for molds and yeasts (ISO 2008); and ISO 15214:1998 for lactic acid bacteria (ISO 1998).

### 2.5 Sensory Analysis

The sensory evaluation protocol was approved by the Research Ethics Committee (CEP) of the Universidade Estadual de Campinas (CAAE number: 64086122.3.0000.5404). All participants signed the Informed Consent Form (ICF), agreeing to voluntarily participate in the tests, as required by Resolution no. 466/2012 of the National Health Council.

Based on the instrumental results, the formulations selected for sensory evaluation were S, F4, F5, and F6 (Table 3). One sample from each formulation was sliced to a thickness ranging from 1.0 to 1.2 mm and subsequently rolled and presented coded to the evaluators. Two slices per formulation were served monadically, following a completely randomized block design. The evaluation ballot comprised:

- Hedonic scale: aiming to evaluate overall liking of the samples, ranging from *extremely disliked* (1) to *extremely liked* (9).
- JAR (just‐about‐right) scale: used to assess the consistency/firmness and saltiness of the samples. Participants rated the intensity of these characteristics on a 7‐point scale ranging from 1 (*much less than ideal*) to 7 (*much more than ideal*), with 4 representing the ideal. For consistency/firmness, they were instructed to manipulate the slice with their hands before tasting it and feeling its consistency/bite resistance to assign a score to the product, whereas for saltiness, they were instructed to simply taste the slice and assess how salty it was to them.
- CATA: Twenty hedonic terms related to the product were presented, and the participants were asked to check all the terms applied as appropriate to describe the cooked ham. The terms were selected based on the description of attributes for cooked ham and turkey ham found in previous studies (Henrique et al. 2015; Mathias 2008; Slongo 2008), representing attributes of appearance, color, flavor, taste, and texture of cooked ham.

**TABLE 3:** Texture parameters (TPA) of cooked hams for each formulation (*n* = 9).

Formulation^a^	Force (N)	Hardness (N)	Elasticity	Cohesiveness	Chewiness	Resilience
S	67.75 ± 9.21^b^	87.70 ± 8.61^b^	0.205 ± 0.010^ab^	0.172 ± 0.010^c^	307.15 ± 24.12^c^	0.055 ± 0.014^d^
C	51.87 ± 8.12^b^	67.20 ± 8.08^c^	0.176 ± 0.004^c^	0.143 ± 0.020^d^	177.59 ± 45.98^d^	0.041 ± 0.008^e^
F1	94.97 ± 10.85^a^	108.14 ± 9.99^a^	0.194 ± 0.009^b^	0.209 ± 0.016^b^	446.88 ± 54.57^b^	0.069 ± 0.007^bc^
F2	95.65 ± 10.98^a^	111.47 ± 11.38^a^	0.196 ± 0.014^b^	0.209 ± 0.017^b^	445.44 ± 77.91^b^	0.063 ± 0.010^bcd^
F3	57.67 ± 8.64^b^	78.05 ± 11.31b^c^	0.222 ± 0.016^a^	0.183 ± 0.025^c^	323.65 ± 86.09^c^	0.059 ± 0.012^cd^
F4	102.95 ± 18.04^a^	119.46 ± 16.57^a^	0.199 ± 0.017^b^	0.216 ± 0.015^b^	513.68 ± 130.27^ab^	0.073 ± 0.004^ab^
F5	109.49 ± 3.07^a^	120.32 ± 6.03^a^	0.207 ± 0.003^ab^	0.238 ± 0.013^a^	590.45 ± 49.48^a^	0.082 ± 0.007^a^
F6	55.77 ± 3.37^b^	66.60 ± 5.44^c^	0.141 ± 0.003^d^	0.102 ± 0.005^e^	326.59 ± 62.54^c^	0.055 ± 0.004^d^

*Note*: Data are presented as mean ± standard error. Means of formulations followed by the same letter, in the column, do not differ from each other using the Tukey test at a 5% probability level.

^a^ Formulations: S = standard formulation; C = reduced‐sodium control; F1 = reduced sodium content with addition of 0.045% ACTIVE BFB*; F2 = reduced sodium content with addition of 0.045% ACTIVE BFB; F3 = reduced sodium content with addition of 1.00% SALT ANSWER M; F4 = reduced sodium content with addition of 0.045% ACTIVA BFB and 1.00% SALT ANSWER M; F5 = reduced sodium content with addition of 0.35% KCl, 0.045% ACTIVA BFB, and 0.25% SALT ANSWER M; F6 = reduced sodium content with addition of 0.35% KCl, 0.0225% ACTIVA BFB, and 0.25% SALT ANSWER M.

The affective sensory test was performed for the four selected samples in the same session. In total, 123 consumers were recruited at the University of Campinas over three consecutive days. They were invited to come to the Laboratory of Studies on Food and Society at the Faculty of Food Engineering, which is fully set up for sensory evaluations.

### 2.6 Cost Estimation for Preparing Each Formulation

To estimate the cost increase associated with replacing sodium chloride, a cost analysis of each of the formulations was conducted based on the price of each ingredient in the formulations. The meat price was based on the Intercarnes Newsletter NR7332 São Paulo (November 1, 2023), referring to boneless industrial pork ham. For the other ingredients, the reference price was given by the respective suppliers.

### 2.7 Statistical Analysis

The study followed a completely randomized design with eight formulations (S, C, F1, F2, F3, F4, F5, and F6). Each formulation was produced in independent 5‐kg batches under identical industrial processing conditions. Due to operational limitations of the pilot plant and the industrial nature of the process, only one production batch was manufactured for each formulation. Physicochemical analyses were performed in triplicate, while texture, color, and expressible water determinations were obtained from multiple subsamples collected from different portions of each cooked ham.

For expressible water, data were analyzed using a two‐way analysis of variance (ANOVA), considering treatment and storage time as fixed effects, as well as their interaction. For physicochemical, technological, and texture parameters, treatment was considered the main fixed effect. When significant differences were detected (*p* ≤ 0.05), means were compared using Tukey's multiple comparisons test. Statistical analyses were performed using IBM SPSS Statistics software, while sensory data were analyzed using XLSTAT software. The results were interpreted as comparisons between formulations produced under the evaluated processing conditions.

Penalty analysis (Lawless and Heymann 2010) was performed on the overall liking and JAR data. First, overall liking ratings were separated into three groups regarding JAR data: ideal, less intense, and more intense than the ideal. Subsequently, the ideal mean rating was subtracted from the other two groups’ mean rating. Finally, the differences between the ideal and the less or more intense mean ratings were statistically compared.

For the CATA data, the frequency of selection for each term and each sample was calculated by creating a contingency table, which was then submitted to correspondence analysis. Statistical analysis was performed using XLStat Sensory (www.xlstat.com) and RedJade Sensory Software.

## 3 Results and Discussion

### 3.1 Physical, Chemical, and Physicochemical Characterization of the Formulations

#### 3.1.1 Approximate Composition

The proximate composition of all formulations met the technical requirements for identity and quality of cooked ham described in SDA Ordinance No. 765 of MAPA, including the moisture/protein ratio, which was below 4.8 in all formulations (Table 4). The results for sodium content indicated a reduction of approximately 30%, which was also reflected in the lower ash content. Similar results were reported by Silva (2020), Delgado‐Pando et al. (2018), Campagnol et al. (2017), and Atilgan and Kilic (2017), owing to the direct relationship between sodium reduction and the reduced availability of inorganic salts and chlorides.

**TABLE 4:** Approximate composition (% content) for the different cooked ham formulations, as well as the respective calculated values of the moisture/protein ratio and sodium amount (mg/100 g).

Formulation^a^	Carbohydrate(%)	Protein (%)	Fat(%)	Ashes(%)	Moisture(%)	Moisture/protein ratio	Sodium amount (mg/100 g)
S	0.9	16.4	1.6	3.2	77.9	4.7	863
C	0.6	17.4	1.6	2.4	78.0	4.5	581
F1	0.7	16.6	1.7	2.5	78.5	4.7	602
F2	0.9	18.4	2.6	2.5	75.6	4.1	606
F3	1.1	16.7	1.9	2.7	77.6	4.6	609
F4	0.4	17.1	2.4	2.8	77.3	4.5	633
F5	0.5	16.7	1.5	2.9	78.4	4.7	633
F6	0.3	17.8	1.8	2.9	77.2	4.3	734

^a^ Formulations: S = standard formulation; C = reduced‐sodium control; F1 = reduced sodium content with addition of 0.045% ACTIVE BFB*; F2 = reduced sodium content with addition of 0.045% ACTIVE BFB; F3 = reduced sodium content with addition of 1.00% SALT ANSWER M; F4 = reduced sodium content with addition of 0.045% ACTIVA BFB and 1.00% SALT ANSWER M; F5 = reduced sodium content with addition of 0.35% KCl, 0.045% ACTIVA BFB, and 0.25% SALT ANSWER M; F6 = reduced sodium content with addition of 0.35% KCl, 0.0225% ACTIVA BFB, and 0.25% SALT ANSWER M.

#### 3.1.2 Physicochemical and Technological Parameters of Cooked Hams

During high‐temperature cooking, proteins denature and have a lower capacity to retain water within their structures, thus causing evaporation, loss due to cooking, and, consequently, shrinkage (Wen et al. 2022). The weight loss identified by Pietrasiki and Gaudette (2014), when reducing the salt content in cooked ham from 2% to 1.2%, was almost twice as great. Salt reduction significantly increased cooking loss, indicating reduced water‐holding capacity and lower water retention within the protein matrix (Table S2). The observed increase in cooking loss after NaCl reduction is likely related to the lower ionic strength, which impairs myofibrillar protein solubilization and decreases water‐binding capacity (T. K. Kim et al. 2021). Similar effects have been reported in reduced‐sodium cooked ham formulations (Atilgan and Kilic 2017; Silva 2020). However, the addition of 1.0% SALT ANSWER M, either alone or combined with transglutaminase, as well as the combination of 0.25% SALT ANSWER M and 0.045% ACTIVA BFB, reversed this effect, resulting in cooking losses comparable to the standard formulation (no significant differences among treatments S, F3, F4, and F5). The improved yield observed in F3, F4, and F5 may be associated with enhanced protein–protein interactions and the formation of a more cohesive gel network during thermal processing. Transglutaminase promotes covalent cross‐linking between myofibrillar proteins, whereas potassium‐based salt replacers can contribute to protein extraction and water immobilization, partially compensating for the reduction in NaCl concentration. As a result, less free water is expelled during cooking, leading to lower cooking losses and improved processing efficiency (Dong et al. 2023).

It is important to emphasize that these variations in cooking loss had no clear relationship with pH or aw (Table 5). These findings indicate that the differences in cooking loss were primarily associated with modifications in protein functionality rather than changes in pH or water activity. The narrow pH range observed among formulations was insufficient to substantially alter protein net charge or water‐binding properties, reinforcing the importance of ionic strength and protein interactions as the main drivers of yield differences (T. K. Kim et al. 2021).

**TABLE 5:** Cooking weight loss values (%PLC), pH, and water activity (*a*_w_) of cooked hams immediately after cooling, 2 days after cooking.

Formulation^a^	%PLC	pH	*a*_w_
S	5.2 ± 0.2^e^	6.27 ± 0.02^cd^	0.976 ± 0.005^d^
C	8.8 ± 0.3^bc^	6.33 ± 0.02^ab^	0.981 ± 0.003^cd^
F1	6.9 ± 0.5^d^	6.33 ± 0.01^ab^	0.991 ± 0.005^a^
F2	15.2 ± 2.4^a^	6.28 ± 0.02^cd^	0.987 ± 0.002^abc^
F3	3.7 ± 0.7^e^	6.31 ± 0.04^abc^	0.983 ± 0.003^bc^
F4	5.6 ± 2.2^e^	6.29 ± 0.04^cd^	0.986 ± 0.003^abc^
F5	5.3 ± 0.4^de^	6.25 ± 0.02^d^	0.986 ± 0.002^abc^
F6	10.7 ± 0.9^b^	6.35 ± 0.01^a^	0.988 ± 0.004^ab^

*Note*: Data are presented as mean ± standard error. Means of formulations followed by the same letter, in the column, do not differ from each other using the Tukey test at a 5% probability level.

^a^ Formulations: S = standard formulation; C = reduced‐sodium control; F1 = reduced sodium content with addition of 0.045% ACTIVE BFB*; F2 = reduced sodium content with addition of 0.045% ACTIVE BFB; F3 = reduced sodium content with addition of 1.00% SALT ANSWER M; F4 = reduced sodium content with addition of 0.045% ACTIVA BFB and 1.00% SALT ANSWER M; F5 = reduced sodium content with addition of 0.35% KCl, 0.045% ACTIVA BFB, and 0.25% SALT ANSWER M; F6 = reduced sodium content with addition of 0.35% KCl, 0.0225% ACTIVA BFB, and 0.25% SALT ANSWER M.

**FIGURE 1:**
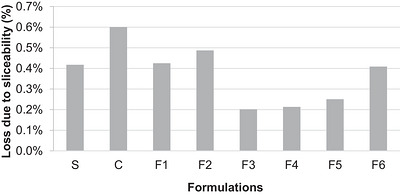
Sliceability loss values (%) in each formulation. Formulations: S = standard formulation; C = reduced‐sodium control; F1 = reduced sodium content with addition of 0.045% ACTIVE BFB*; F2 = reduced sodium content with addition of 0.045% ACTIVE BFB; F3 = reduced sodium content with addition of 1.00% SALT ANSWER M; F4 = reduced sodium content with addition of 0.045% ACTIVA BFB and 1.00% SALT ANSWER M; F5 = reduced sodium content with addition of 0.35% KCl, 0.045% ACTIVA BFB, and 0.25% SALT ANSWER M; F6 = reduced sodium content with addition of 0.35% KCl, 0.0225% ACTIVA BFB, and 0.25% SALT ANSWERTM M.

Regarding sliceability losses (Figure 1), a positive association was observed between slicing losses and cooking losses, indicating that both parameters were strongly influenced by the ability of the protein matrix to retain water and maintain structural integrity during thermal processing (Warmer 2023). This relationship is supported by the fact that the reduced‐sodium control (C), which exhibited a cooking loss of 8.8%, showed substantially poorer technological performance than the standard formulation (5.2%). The increase in sliceability losses after sodium reduction can be attributed to the lower ionic strength of the formulation, which reduces myofibrillar protein extraction and limits the formation of a continuous and cohesive gel network (Chen et al. 2025). The reduction in sliceability performance is particularly relevant from an industrial perspective because slicing losses directly affect commercial yield, product appearance, and process efficiency (Steen et al. 2020). Products with poor structural integrity tend to generate a greater proportion of fragments and irregular slices, reducing their suitability for retail packaging and increasing economic losses during processing (Neves et al. 2020). In the present study, the addition of 1.0% SALT ANSWER M (F3) or the combination of 0.25% SALT ANSWER M and 0.045% ACTIVA BFB (F5) reduced sliceability losses to approximately half of those observed in the standard formulation, demonstrating a substantial improvement in structural stability. This effect can be attributed, at least in part, to the action of transglutaminase, which promotes covalent cross‐linking between glutamine and lysine residues in muscle proteins, strengthening the protein network and increasing resistance to mechanical stress during slicing (Vasić et al. 2023). The TPA supports this interpretation, as formulations containing 0.045% transglutaminase showed markedly higher cohesiveness and chewiness than the reduced‐sodium control. Cohesiveness increased from 0.143 in treatment C to 0.216 and 0.238 in F4 and F5, respectively, while chewiness increased from 177.59 to 513.68 and 590.45 N. These improvements indicate the formation of a more cohesive and resilient protein matrix, which likely contributed to the lower sliceability losses observed (Verdu et al. 2025). Similar effects of transglutaminase on gel strength, cohesiveness, and structural stability have been reported in reduced‐sodium meat products (Ren et al. 2024; J. Zhang et al. 2025). Interestingly, formulations containing meat seasoning showed the lowest sliceability losses. Beyond its flavoring role, this ingredient may have contributed to improved water retention and protein–protein interactions through the presence of potassium salts, yeast extract, and other functional compounds (Yu et al. 2025). The lower cooking losses observed in F4 and F5 support this interpretation, suggesting a synergistic effect between seasoning components and protein network stabilization, which helped maintain product integrity despite NaCl reduction.

The color parameters were evaluated 5 days after manufacturing (Table 6). The sodium‐reduced control and the formulations with reduced salt containing only transglutaminase (F1, F2) were the lightest (lowest luminosity), while all the others had no significant (*p* > 0.05) difference in terms of luminosity compared to the standard product. For the a* chromaticity index parameter (red), there was no statistical difference between any of the treatments, while b* (yellow) tended to increase for NaCl‐reduced products without meat flavoring. In other studies, sodium reduction from 2% to 1.2% in cooked hams yielded lighter, yellower, and less reddish products (Pietrasik and Gaudette 2014). According to the results of global color change (ΔE*_ab_), considering a color discrimination threshold for ΔE*_ab_ close to 2.3 according to Mokrzycki and Tatol (2011), there were no differences visible to the human eye between the standard formulation and treatments F4–F6, while F2 had the greatest color difference in relation to the standard formulation.

**TABLE 6:** Objective color parameters of cooked hams after cooling.

Formulation^a^	Color parameter			
	L*	a*	b*	ΔE*_ab_
S	56.3 ± 0.8^d^	12.4 ± 0.4^a^	6.6 ± 0.2^de^	0
C	59.1 ± 0.3^abc^	12.0 ± 0.3^a^	7.7 ± 0.1^abc^	3.0
F1	59.7 ± 1.2^ab^	11.4 ± 0.5^a^	8.0 ± 0.6^a^	3.8
F2	61.0 ± 0.2^a^	12.2 ± 0.4^a^	7.9 ± 0.1^ab^	3.0
F3	58.9 ± 1.1^cd^	11.3 ± 0.8^a^	7.7 ± 0.1^ab^	0.7
F4	56.9 ± 1.2^cd^	12.4 ± 0.2^a^	6.9 ± 0.2^cd^	0.9
F5	57.0 ± 1.6^bcd^	12.3 ± 0.6^a^	7.2 ± 0.2^bcd^	4.8
F6	56.7 ± 0.1^cd^	12.7 ± 0.1^a^	6.0 ± 0.1^e^	0.8

*Note*: Data are presented as mean ± standard error. Means of formulations followed by the same letter, in the column, do not differ from each other using the Tukey test at a 5% probability level.

^a^ Formulations: S = standard formulation; C = reduced‐sodium control; F1 = reduced sodium content with addition of 0.045% ACTIVE BFB*; F2 = reduced sodium content with addition of 0.045% ACTIVE BFB; F3 = reduced sodium content with addition of 1.00% SALT ANSWER M; F4 = reduced sodium content with addition of 0.045% ACTIVA BFB and 1.00% SALT ANSWER M; F5 = reduced sodium content with addition of 0.35% KCl, 0.045% ACTIVA BFB, and 0.25% SALT ANSWER M; F6 = reduced sodium content with addition of 0.35% KCl, 0.0225% ACTIVA BFB, and 0.25% SALT ANSWER M.

Texture is an important quality parameter in cooked ham because it directly affects slicing performance (Perondi 2015). According to the TPA (Table 3), salt reduction negatively affected several textural attributes, including force, hardness, springiness, cohesiveness, chewiness, and resilience. These results are consistent with previous studies reporting that sodium reduction weakens myofibrillar protein extraction and gel formation, resulting in softer and less cohesive meat matrices (Lee et al. 2023; Di Domenico et al. 2024).

The treatments containing 0.045% transglutaminase (F1, F2, F4, and F5) exhibited significantly higher (*p* < 0.05) force and hardness values than the reduced‐sodium control, demonstrating that the enzyme effectively compensated for the structural weakening caused by NaCl reduction (Gao et al. 2025). In several cases, textural values were comparable to or even exceeded those of the standard formulation, indicating the formation of a stronger protein network. Similar improvements have been reported in reduced‐sodium meat products and protein‐based systems containing microbial transglutaminase (Amirdivani et al. 2018; H. Kim et al. 2022; J. Zhang et al. 2025).

The positive effect of transglutaminase is attributed to its ability to catalyze covalent cross‐links between glutamine and lysine residues in muscle proteins, strengthening the gel matrix and enhancing its resistance to deformation (Ren et al. 2024; Li et al. 2026). Consequently, treatments containing transglutaminase showed increased cohesiveness, chewiness, and resilience, reflecting improved structural integrity during mastication. In addition, the incorporation of 1.0% meat seasoning restored springiness and other texture parameters to levels statistically like those of the standard formulation, suggesting a complementary contribution to water retention and protein functionality (Ayuso et al. 2025). Overall, these findings indicate that transglutaminase was the main factor responsible for recovering and enhancing the textural quality of reduced‐sodium cooked ham formulations.

An additional comparison between the two transglutaminase preparations evaluated in this study revealed distinct technological responses. Formulations containing ACTIVA BFB* generally exhibited higher force, hardness, cohesiveness, chewiness, and resilience values than formulations containing ACTIVA BFB, suggesting a stronger reinforcement of the protein matrix under the conditions evaluated. This behavior suggests a greater apparent cross‐linking effect promoted by ACTIVA BFB*, resulting in a more rigid gel network (H. Kim et al. 2022). However, this stronger structure was not necessarily associated with superior product performance. Sensory results showed that F4 was frequently perceived as excessively firm, whereas F5 exhibited a more balanced texture profile and higher consumer acceptance. Furthermore, F5 exhibited one of the lowest expressible water values among the reduced‐sodium formulations, demonstrating that effective water retention could be achieved without excessive matrix rigidity. Although enzymatic activity was not directly measured, the combined texture, water retention, and sensory results suggest that ACTIVA BFB* promoted a stronger apparent technological effect, whereas ACTIVA BFB provided a more favorable balance between structural reinforcement, water retention, and sensory quality. These findings indicate that the selection of the transglutaminase preparation may be as important as its concentration in the development of reduced‐sodium cooked ham formulations.

In this study, water retention was evaluated as expressible water after 2, 15, and 30 days of storage (Table 7). As expected, reducing NaCl content increased water loss throughout storage, confirming the detrimental effect of sodium reduction on the water‐holding capacity of cooked ham. However, this effect was partially or completely mitigated by the incorporation of functional ingredients commonly used in reduced‐sodium meat products, such as phosphates, nonmeat proteins, stabilizers, flavor enhancers, potassium chloride, and transglutaminase, which contribute to maintaining texture, water retention, and overall product quality (Fang and Zhu 2025).

**TABLE 7:** Expressible water (%) in cooked hams after 2, 15, and 30 days of production.

Formulation^a^	Days after production			Interaction
	2	15	30	Treatments versus days of production
S	25.1 ± 3.4^cA^	21.7 ± 1.9^cB^	25.47 ± 2.5^cA^	*
C	35.4 ± 1.9^aA^	32.20 ± 4.2^aB^	32.99 ± 3.9^aAB^	*
F1	29.6 ± 3.5^bA^	27.40 ± 4.0^bAB^	26.30 ± 2.2^cB^	*
F2	31.8 ± 3.9^bA^	28.62 ± 3.0^bB^	29.87 ± 3.9^bA^	*
F3	23.9 ± 2.5^cA^	21.92 ± 1.6^cB^	24.40 ± 2.1^cdA^	*
F4	22.1 ± 2.4^cA^	19.55 ± 2.1^cB^	21.47 ± 1.3^deB^	*
F5	23.4 ± 2.2^cA^	20.13 ± 2.3^cA^	19.54 ± 4.2^eB^	*
F6	30.0 ± 3.5^bB^	32.30 ± 4.3^aA^	31.46 ± 3.1^abAB^	*

*Note*: Data are presented as mean ± standard error. Means of formulations followed by the same letter, in the column, do not differ from each other using the Tukey test at a 5% probability level. Means of formulations followed by the same capital letter in the line do not differ from each other using Tukey's test at a 5% probability level.

^a^ Formulations: S = standard formulation; C = reduced‐sodium control; F1 = reduced sodium content with addition of 0.045% ACTIVE BFB*; F2 = reduced sodium content with addition of 0.045% ACTIVE BFB; F3 = reduced sodium content with addition of 1.00% SALT ANSWER M; F4 = reduced sodium content with addition of 0.045% ACTIVA BFB and 1.00% SALT ANSWER M; F5 = reduced sodium content with addition of 0.35% KCl, 0.045% ACTIVA BFB, and 0.25% SALT ANSWER M; F6 = reduced sodium content with addition of 0.35% KCl, 0.0225% ACTIVA BFB, and 0.25% SALT ANSWER M.

*Significant at the 5% probability level (*p* < 0.05).

A significant interaction between treatment and storage time was observed. Formulations S, F2, F3, and F4 exhibited greater water loss after 15 days of storage, whereas F5 reached its highest expressible water values after 30 days. The reduced‐sodium control (C) consistently showed the highest water release, although no significant (*p* > 0.05) differences were observed among storage times. The most effective treatments for improving water retention were those containing 1.0% meat flavoring (F3 and F4) and the formulation combining 0.25% meat flavoring, 0.35% KCl, and 0.045% transglutaminase (F5). In contrast, the formulation containing 0.0225% transglutaminase (F6) did not improve water retention during storage, indicating that this concentration was insufficient to counterbalance the effects of sodium reduction.

Overall, the results demonstrate that sodium reduction negatively affected water‐holding capacity, whereas the incorporation of meat flavoring, KCl, and transglutaminase partially or completely compensated for this effect. The significant treatment × storage interaction indicates that the effectiveness of these ingredients varied throughout storage, particularly in formulations containing transglutaminase, highlighting the dynamic nature of water retention in reduced‐sodium cooked hams (Zhu et al. 2025; Kieliszek 2026).

Based on the results presented so far, sensory evaluation was proposed for formulations F4, F5, and the standard formulation (S) as a reference product. We also proposed formulation F6 (instrumental data analyzed after sensory analyses), considering that an intermediate transglutaminase load would result in better acceptance. To confirm compliance with national legislation, microbiological assays were performed on those samples, as presented next.

### 3.2 Microbiological Analysis of Cooked Hams

The cooked hams selected for sensory evaluation (S, F4, F5, and F6) were analyzed after 4, 15, and 33 days of production (Table S2). All treatments were negative for *Salmonella* spp., *E. coli*, and *C. perfringens*, and the coagulase‐positive *Staphylococcus* counts remained within the limits established by legislation. Although not required by Brazilian legislation, lactic acid bacteria were quantified because they are spoilage agents in thermally processed meat products (Dušková et al. 2016). The counts were always <10 CFU/g, except for treatment F5 after 33 days. During the final analysis period (33 days after production), all formulations showed clear signs of deterioration, including syneresis, sliminess, and changes in color and odor, indicating that they were no longer suitable for consumption. However, the sensory analyses were performed using freshly prepared samples (<12 days).

### 3.3 Sensory Analysis of the Cooked Hams

The results of the acceptance test are presented in Table 8. The standard formulation was the most accepted, followed by F6, F5, and F4, with a significant difference between F6 and F4 (*p* < 0.05). F5 did not differ significantly from either.

**TABLE 8:** Mean acceptance ratings of formulations S, F4, F5, and F6.

Formulations^a^	S	F4	F5	F6
Acceptance	7.4 ± 1.4^a^	6.4 ± 1.9^c^	6.5 ± 1.9^bc^	6.9 ± 1.7^b^

Means ± standard error. Means of formulations followed by the same letter, in the line, do not differ from each other using the Tukey test at a 5% probability level.

^a^ Formulations: S = standard formulation; F4 = reduced sodium content with addition of 0.045% ACTIVA BFB and 1.00% SALT ANSWER M; F5 = reduced sodium content with addition of 0.35% KCl, 0.045% ACTIVA BFB, and 0.25% SALT ANSWER M; F6 = reduced sodium content with addition of 0.35% KCl, 0.0225% ACTIVA BFB, and 0.25% SALT ANSWER M.

In general, consumers preferred the standard formulation (*p* < 0.05), followed by samples F6 and F5. The proposed sodium reduction had a negative impact on ham acceptance, particularly for the F4 sample. On the other hand, it is important to note that there was no rejection of the reformulated samples, as the acceptance rate was higher than 6.0 on the hedonic scale (between lightly and moderately liked).

**FIGURE 2:**
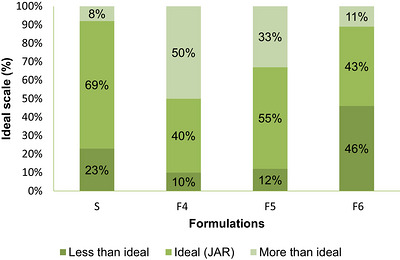
Ideal scale for cooked hams—texture/consistency (S, F4, F5, and F6). Formulations: S = standard formulation; F4 = reduced sodium content with addition of 0.045% ACTIVA BFB and 1.00% SALT ANSWER M; F5 = reduced sodium content with addition of 0.35% KCl, 0.045% ACTIVA BFB, and 0.25% SALT ANSWER M; F6 = reduced sodium content with addition of 0.35% KCl, 0.0225% ACTIVA BFB, and 0.25% SALT ANSWER M.

**FIGURE 3:**
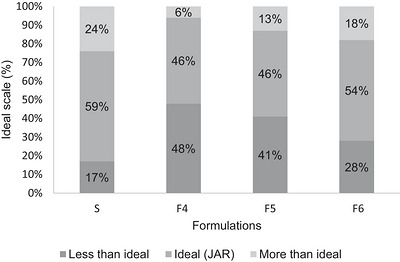
Ideal scale for cooked hams—salty taste (S, F4, F5, and F6). Formulations: S = standard formulation; F4 = reduced sodium content with addition of 0.045% ACTIVA BFB and 1.00% SALT ANSWER M; F5 = reduced sodium content with addition of 0.35% KCl, 0.045% ACTIVA BFB, and 0.25% SALT ANSWER M; F6 = reduced sodium content with addition of 0.35% KCl, 0.0225% ACTIVA BFB, and 0.25% SALT ANSWER M.

Figures 2 and 3 show the JAR ideal scale (7 points) for the attributes of texture/consistency and salty taste. None of the treatments achieved an ideal acceptance of greater than 70%. Compared to the standard formulation (S), salt reduction with the addition of 0.045% transglutaminase and 1% meat flavoring (F4) resulted in a higher percentage of ratings of a more than ideal texture and a less than ideal salty taste, while the reduction of meat flavoring to 0.25% and the concomitant inclusion of 0.35% KCl (F5) improved the texture (reestablishing the same percentage of ideal texture as the standard), but did not significantly impact the salty taste. Compared to this treatment, the halving of transglutaminase (F6) caused the texture classification to drop to less than ideal, without changing the “ideal” classification for salty taste. Formulations F4 and F5 had the highest results for more than ideal texture/consistency, at 45% and 30%, respectively, demonstrating the action of transglutaminase and resulting in a reduction, though not as pronounced as F6, which had 40% texture/consistency below the ideal. In terms of saltiness, the large percentages below the ideal (48% and 50%, respectively) indicate that the treatments’ salty taste was still less than ideal.

**FIGURE 4:**
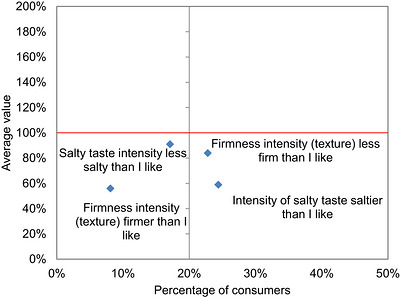
Penalty analysis for the standard formulation (S).

**FIGURE 5:**
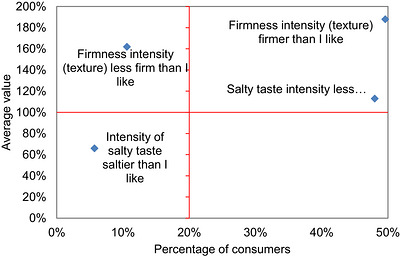
Penalty analysis for the standard formulation (S).

**FIGURE 6:**
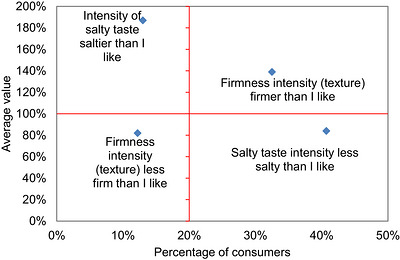
Penalty analysis for the F5 formulation.

**FIGURE 7:**
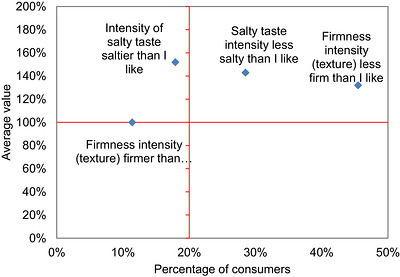
Penalty analysis for the F6 formulation.

Penalty analysis provides the attributes evaluated according to the treatments, starting with the standard formulation S (Figure 4). For formulation F4 (Figure 5), 49.6% of consumers penalized the texture as too firm, and 48% penalized the sample's lack of saltiness. Considering formulation F5 (Figure 6), it was penalized for being “firmer than I like” and “less salty than I like” by, respectively, 32.5% and 40.7% of consumers. Formulation F6 (Figure 7) was penalized for being less firm (for 45.5% of consumers) and less salty (for 28.5% of consumers). These results are consistent with the data obtained with JAR (Figure 2).

The terms weak pink color, pale, and little salt had a significantly negative impact on the average global acceptance of the treatments. These two attributes, pale color and salty taste intensity, are of great relevance and need to be reworked in new developments.

**FIGURE 8:**
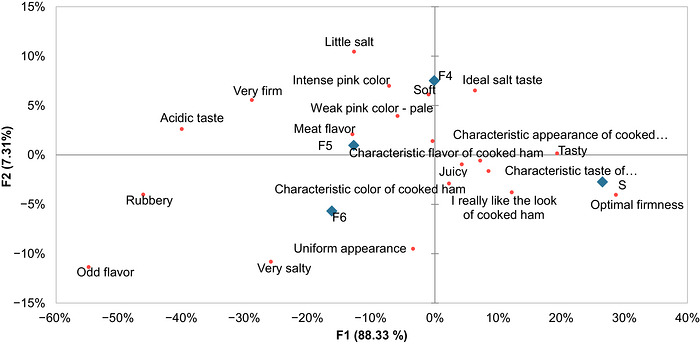
Correspondence analysis (CA) map based on CATA term frequencies for cooked ham formulations. Dimension F1 (88.33%) mainly separated samples according to overall sensory acceptance and typical cooked ham characteristics, with positive values associated with attributes such as juicy, tasty, characteristic appearance, and optimal firmness. Negative F1 values were associated with undesirable attributes, including rubbery texture, odd flavor, acidic taste, and low salt perception. Dimension F2 (7.31%) was mainly related to differences in texture perception, salty taste intensity, and color attributes among reduced‐sodium formulations.

Correspondence analysis was performed on the CATA data, and the first two extracted dimensions accounted for 95.64% of the variance in the experimental data, with 88.3% and 7.31% for the first and second dimensions, respectively. Figure 8 shows the terms used to describe the treatments in the first two dimensions of the multiple factor analysis on the CATA data, considering the acceptance and representation of the four cooked ham treatments. F5 and F6 were associated with similar sensory characteristics located at negative values of the first dimension. F4 was located at intermediate values of the first dimension and positive values of the second dimension, and finally, S was located at a positive value of the first dimension. The standard treatment was associated with aroma, flavor, and the characteristic color of cooked ham, juicy, tasty, ideal firmness, and the statement, “I really like the appearance of cooked ham.” F4 was associated with intense pink color, softness, and ideal salty taste, and F5 was associated with meat flavor and the characteristic appearance of cooked ham.

The sensory results indicate that, although transglutaminase contributed to improving texture and KCl partially compensated for sodium reduction, none of the reformulated treatments achieved an ideal balance between texture and salty taste. The high percentages of consumers classifying the products as less salty than ideal highlight the challenge of maintaining flavor perception in reduced‐sodium cooked ham. Future formulation strategies could include the use of flavor enhancers such as yeast extracts, umami compounds, amino acids, or natural flavoring systems capable of enhancing salt perception without increasing sodium content (Vidal et al. 2020). In addition, optimizing the concentration of transglutaminase may help avoid excessive firmness, which was identified as a major driver of consumer penalties in formulations F4 and F5. From an industrial perspective, these findings demonstrate that technological improvements alone are insufficient to ensure consumer acceptance and that successful sodium‐reduction strategies must simultaneously address flavor, texture, and appearance attributes. Further studies evaluating different combinations of salt replacers, flavor enhancers, and texture‐modifying ingredients are warranted to achieve formulations that meet both public health recommendations and consumer expectations.

### 3.4 Cost Comparison

The use of new technologies to maintain technological characteristics that would be lost with the reduction of sodium chloride would have an estimated impact of around 11% on the final cost ($/kg) (Table S3).

Comparing the costs between F4 and F5, reducing the dosage of meat seasoning from 1.0% to 0.25% brought a cost reduction of 3.7 percentage points, proving to be efficient with the potassium chloride supplement. However, when reducing the transglutaminase dosage by 50%, from F5 to F6, an optimal dosage point has not yet been obtained, and therefore, more tests need to be carried out. But the predicted costs for treatments F5 and F6 are those recommended so far.

## 4 Conclusions

The combination of transglutaminase, KCl, and meat seasoning improved several technological properties of reduced‐sodium cooked ham, demonstrating its potential as a sodium‐reduction strategy. Despite the technological improvements, the reduced‐sodium formulations did not achieve sensory equivalence to the standard product, mainly due to lower saltiness, rubbery texture, and paler pink color. Future studies should focus on optimizing transglutaminase levels, enhancing salty taste perception, and improving product color to increase consumer acceptance. Although the study was conducted using a single industrial batch per treatment, the results provide valuable information under real processing conditions and may support future sodium‐reduction strategies for healthier meat products. From a practical perspective, these findings may assist meat processors in developing reduced‐sodium cooked ham formulations with acceptable technological performance. The results may also support public health and regulatory initiatives aimed at reducing sodium intake. Growing consumer interest in healthier foods suggests potential market opportunities for reformulated meat products. However, maintaining sensory quality remains a key challenge. Therefore, further optimization is necessary to ensure consumer acceptance and commercial success.

## Author Contributions

**Simoni Cristina Mendes Cardim**: conceptualization, methodology, formal analysis, writing – original draft, writing – review and editing, data curation, investigation. **Gilmar Freire da Costa**: methodology, formal analysis, writing – original draft, writing – review and editing, investigation. **Marise Aparecida Rodrigues Pollonio**: conceptualization, resources, writing – review and editing, validation. **Jorge Herman Behrens**: conceptualization, validation, data curation, writing – review and editing, resources. **Juliano Lemos Bicas**: conceptualization, writing – review and editing, project administration, supervision, resources, funding acquisition.

## Conflicts of Interest

Juliano Lemos Bicas was financed by the Coordination of Higher Education Personnel Improvement and the National Council for Scientific and Technological Development. Simoni Cristina Mendes Cardim worked at Ajinomoto (Brazil) during the execution of this project. The authors declare no other conflicts of interest.

## Supporting information




**Supplementary Materials**: jfds71358‐sup‐0001‐SuppMat.docx
