# Supplementary material for: Development of Reduced‐Sodium Cooked Ham Using NaCl Substitutes: Impact on Instrumental and Sensory Properties
Source: J Food Sci. 2026 Aug 15;91(8):e71358. doi: 10.1111/1750-3841.71358 (PMC13476952; doi:10.1111/1750-3841.71358)
Supplement: Supplementary file 1 [file JFDS-91-0-s001.docx]

**Supplementary data**

*S1. Starting materials*

The following starting materials were used to produce the cooked hams: boneless pork shank purchased from the Porco Feliz prime meat shop at Mercado Municipal de São Paulo, table salt (NaCl, Cismal), table sugar (sucrose, Alta Mogiana), soy protein isolate (Solae Supro® 5481P), thickener containing sucrose and carrageenan (8020 PLUS-TX, Adeste), sodium tripolyphosphate and sodium polyphosphate (Curafos F621, ICL), Hungarian III powder containing sodium chloride 93-95%, sodium nitrite 5% and sodium nitrate sodium 1% (Adeste), antioxidant/color fixer composed of sodium erythorbate and sulfite (0964/0028, Duas Rodas), monosodium glutamate (Ajinomoto), cochineal carmine dye (CC-WS-P, Chr Hansen), California 350 ham seasoning containing 91.60% NaCl, 5.10% sucrose, 0.80% MSG, 1.20% garlic powder, 1.20% oil, 0.80% onion powder, 0.30% clove resin, 0.20% cinnamon essential oil (New Max), meat flavor condiment containing potassium chloride, maltodextrin, sugar, vegetable fat, hydrolyzed vegetable protein, salt, yeast extract, white pepper, aroma identical to natural meat, citric acid, monosodium glutamate, disodium inosinate, disodium guanylate, silicon dioxide, tricalcium phosphate and sodium citrate (totalizing 22,15 mg of sodium per gram) (SALT ANSWER^TM^ M, Ajinomoto), and two enzymatic preparations containing transglutaminase obtained from *Streptomyces mobaraense*, called ACTIVA BFB (enzymatic activity 130-240 U/ g and 0.22 mg of sodium/g) and ACTIVA BFB* (enzymatic activity 109-188 U/ g and 0.13 mg of sodium/g). Although ACTIVA BFB* has been discontinued, it was included in this study to evaluate the possible effect of enzyme activity on the final results.

**Supplementary data - Tables and Figure**

Supplementary Table S1- Pressed juiciness (%) in cooked hams interactions: treatments x 2, 15, and 30 days of production.

| FV | GL | SQ | QM | F |
| --- | --- | --- | --- | --- |
| Treatments ( F1 ) | 6 | 6014.47881 | 1002.41313 | 122.5489** |
| Days of production ( F2 ) | 2 | 398.90540 | 199.45270 | 24.3839** |
| Interaction ( F1 x F2 ) | 12 | 174.90274 | 14.57523 | 1.7819* |
| Treatment | 20 | 6588.28695 | 329.41435 | 40.2722** |
| Residual | 294 | 2404.83129 | 8.17970 |  |
| Total | 314 | 8993.11824 |  |  |

**significant at the 1% probability level (*P* <0.01).

*significant at the 5% probability level (*P* <0.05).

FV: Source of variation

GL: Degree of freedom

SQ: Sum of squares

QM: Mean square

Supplementary Table S2 –Values for moldes and yeasts, *Escherichia coli*, coagulase-positive Staphylococci, *Clostridium perfringens* and lactic acid bacteria (CFU/ g) and *Salmonella* spp. (in 25 g) in cooked hams

| Formula-tion | Settlement | Days after preparation | | |
| --- | --- | --- | --- | --- |
|  |  | 4 | 15 | 33 |
| S | Mols and yeasts | 20 | < 10 | 160.000 |
|  | *Escherichia coli* | < 10 | < 10 | < 10 |
|  | Coagulase-positive Staphylococci | < 10 | < 10 | < 100 |
|  | *Clostridium perfringens* | < 10 | < 10 | < 10 |
|  | Lactic acid bacteria | < 10 | < 10 | 20 |
|  | *Salmonella* spp. | Absent | Absent | Absent |
| F4 | Molds and yeasts | < 10 | < 10 | 8.700 |
|  | *Escherichia coli* | < 10 | < 10 | < 10 |
|  | Coagulase-positive Staphylococci | < 10 | < 10 | < 100 |
|  | *Clostridium perfringens* | < 10 | < 10 | < 10 |
|  | Lactic acid bacteria | < 10 | < 10 | < 10 |
|  | *Salmonella* spp. | Absent | Absent | Absent |
| F5 | Mols and yeasts | < 10 | < 10 | 570 |
|  | *Escherichia coli* | < 10 | < 10 | < 10 |
|  | Coagulase-positive Staphylococci | < 10 | < 10 | < 100 |
|  | *Clostridium perfringens* | < 10 | < 10 | < 10 |
|  | Lactic acid bacteria | 30 | < 10 | 310 |
|  | *Salmonella* spp. | Absent | Absent | Absent |
| F6 | Molds and yeasts | < 10 | < 10 | < 10 |
|  | *Escherichia coli* | < 10 | < 10 | < 10 |
|  | Coagulase-positive Staphylococci | < 10 | < 10 | < 100 |
|  | *Clostridium perfringens* | < 10 | < 10 | < 10 |
|  | Lactic acid bacteria | < 10 | < 10 | 10 |
|  | *Salmonella* spp. | Absent | Absent | Absent |

* Formulations: S = Standard formulation;

F4 = reduced sodium content with addition of 0.045% ACTIVA BFB and 1.00% SALT ANSWER^TM^ M;

F5 = reduced sodium content with addition of 0.35% KCl, 0.045% ACTIVA BFB and 0.25% SALT ANSWER^TM^ M;

F6 = reduced sodium content with addition of 0.35% KCl, 0.0225% ACTIVA BFB and 0.25% SALT ANSWER^TM^ M.

Supplementary Table S3 – Cost comparison between treatments of cooked ham with sodium reduction using ACTIVA BFB and SALT ANSWER^TM^ M (Ajinomoto).

| **Ingredient** | **Unitary value ($/kg)** | **Added Percentage (%)** | | | | | | | **Cost impact on formulation ($/kg)** | | | | | | |
| --- | --- | --- | --- | --- | --- | --- | --- | --- | --- | --- | --- | --- | --- | --- | --- |
|  |  | S | F1 | F2 | F3 | F4 | F5 | F6 | S | F1 | F2 | F3 | F4 | F5 | F6 |
| Extra clean pork ham | 2.295 | 62.50 | 62.50 | 62.50 | 62.50 | 62.50 | 62.50 | 62.50 | 1.434 | 1.434 | 1.434 | 1.434 | 1.434 | 1.434 | 1.434 |
| Soy Protein Isolate  (Solae Supro® 548IP) | 2.254 | 2.00 | 2.00 | 2.00 | 2.00 | 2.00 | 2.00 | 2.00 | 0.045 | 0.045 | 0.045 | 0.045 | 0.045 | 0.045 | 0.045 |
| Salt (NaCl) (Cimsal) | 0.111 | 1.00 | 0.30 | 0.30 | 0.24 | 0.24 | 0.28 | 0.28 | 0.001 | 0.000 | 0.000 | 0.000 | 0.000 | 0.000 | 0.000 |
| California 350 ham seasoning  (New Max) | 0.762 | 0.65 | 0.65 | 0.65 | 0.65 | 0.65 | 0.65 | 0.65 | 0.005 | 0.005 | 0.005 | 0.005 | 0.005 | 0.005 | 0.005 |
| Sucrose (Alta Mogiana) | 0.338 | 0.70 | 0.70 | 0.70 | 0.70 | 0.70 | 0.70 | 0.70 | 0.002 | 0.002 | 0.002 | 0.002 | 0.002 | 0.002 | 0.002 |
| Monosodium glutamate (Ajinomoto) | 1.844 | 0.30 | 0.30 | 0.30 | 0.30 | 0.30 | 0.30 | 0.30 | 0.006 | 0.006 | 0.006 | 0.006 | 0.006 | 0.006 | 0.006 |
| Carrageenan (Adeste 8020) | 4.508 | 0.50 | 0.50 | 0.50 | 0.50 | 0.50 | 0.50 | 0.50 | 0.023 | 0.023 | 0.023 | 0.023 | 0.023 | 0.023 | 0.023 |
| Tripolyphosphate and Sodium Polyphosphate (ICL) | 2.869 | 0.15 | 0.15 | 0.15 | 0.15 | 0.15 | 0.15 | 0.15 | 0.004 | 0.004 | 0.004 | 0.004 | 0.004 | 0.004 | 0.004 |
| Color fixer 0964/0028 (Duas Rodas) | 2.756 | 0.50 | 0.50 | 0.50 | 0.50 | 0.50 | 0.50 | 0.50 | 0.014 | 0.014 | 0.014 | 0.014 | 0.014 | 0.014 | 0.014 |
| Hungarian Powder IIIIII (Adeste) | 0.553 | 0.20 | 0.20 | 0.20 | 0.20 | 0.20 | 0.20 | 0.20 | 0.001 | 0.001 | 0.001 | 0.001 | 0.001 | 0.001 | 0.001 |
| Cochineal Carmine Dye (Chr Hansen) | 12.367 | 0.03 | 0.03 | 0.03 | 0.03 | 0.03 | 0.03 | 0.03 | 0.003 | 0.003 | 0.003 | 0.003 | 0.003 | 0.003 | 0.003 |
| Water/ Ace | 0.000 | 31.48 | 32.13 | 32.13 | 31.23 | 31.19 | 31.55 | 31.57 | 0.000 | 0.000 | 0.000 | 0.000 | 0.000 | 0.000 | 0.000 |
| Potassium chloride (KCl) (Aksell) | 1.844 | 0.00 | 0.00 | 0.00 | 0.00 | 0.00 | 0.35 | 0.35 | 0.000 | 0.000 | 0.000 | 0.000 | 0.000 | 0.006 | 0.006 |
| ACTIVA BFB (Ajinomoto)/  ACTIVA BFB* (Ajinomoto) | 90.000 | 0.00 | 0.05 | 0.05 | 0.00 | 0.05 | 0.05 | 0.02 | 0.000 | 0.041 | 0.041 | 0.000 | 0.041 | 0.041 | 0.020 |
| SALT ANSWER^TM^ M (Ajinomoto) | 8.500 | 0.00 | 0.00 | 0.00 | 1.00 | 1.00 | 0.25 | 0.25 | 0.000 | 0.000 | 0.000 | 0.085 | 0.085 | 0.021 | 0.021 |
|  |  | **Total value of each formulation ($/kg)** | | | | | | | 1.538 | 1.578 | 1.578 | 1.622 | 1.663 | 1.606 | 1.585 |
| **Difference from the standard formulation (%)** | | | | | | | | | - | 2.6 | 2.6 | 5.5 | 8.1 | 4.4 | 3.1 |

* Formulations: S = Standard formulation;

F1 = reduced sodium content with addition of 0.045% ACTIVE BFB*;

F2= reduced sodium content with addition of 0.045% ACTIVE BFB;

F3 = reduced sodium content with addition of 1.00% SALT ANSWER^TM^ M;

F4 = reduced sodium content with addition of 0.045% ACTIVA BFB and 1.00% SALT ANSWER^TM^ M;

F5 = reduced sodium content with addition of 0.35% KCl, 0.045% ACTIVA BFB and 0.25% SALT ANSWER^TM^ M;

F6 = reduced sodium content with addition of 0.35% KCl, 0.0225% ACTIVA BFB and 0.25% SALT ANSWER^TM^ M.

**Description of ingredients used**

| Ingredient | Manufactu-rer | Composition | Function |
| --- | --- | --- | --- |
| Soy Protein Isolate Supro® 548IP | Solae | Isolated soy protein | High degree of functionality for  meat products. Allows increase of  yield, better texture, appearance and sliceability. |
| California 350 ham seasoning | New Max | Salt (91.60%), sugar (5.10%), flavor enhancer monosodium glutamate (0.80%), spices (garlic powder: 1.20% and onion powder: 0.80%), and natural flavoring (clove resin oil: 0.30% and cinnamon essential oil: 0.20%). | Based on spices and natural flavorings, used to season, imparting characteristic flavor. |
| Carrageenan (8020) | Adeste | Sugar and carrageenan thickener. | Thickener |
| Tripolyphosphate and Sodium Polyphosphate | ICL | Sodium tripolyphosphate and sodium polyphosphate. | Used in brines for fresh and cooked meats. High capacity of protein extraction.  Facilitates the link between water and meat protein,  reduces loss during baking,  and optimizes the properties of meat during tumbling. |
| Antioxidant 0964/0028 | Duas Rodas | Sugar and sodium erythorbate (INS 316). | Improves the texture, yield, and conservation of meat products. |
| Hungarian Powder III | Adeste | Refined salt (93-95%), Sodium nitrite (5%) and Sodium nitrate (1%). | Preservative |
| Cochineal Carmine Dye | Chr Hansen | Carmine (INS 120), maltodextrin and potassium hydroxide (INS 525). | Water-soluble powder dye, produced from calcium-aluminum acid lacquer carmine, obtained from the dry body of the insect *Dactylopius coccus* costa, which is dissolved in an aqueous alkali solution, added with maltodextrin and dehydrated in “spray-dryer” type equipment. |
| Activa BFB/ BFB* | Ajinomoto | Monoammonium glutamate, maltodextrin, vegetable oil and transglutaminase. | Enzyme preparation with transglutaminase obtained from *Streptomyces mobaraense*. |
| Salt Answer ^TM^ | Ajinomoto | Potassium chloride, maltodextrin, sugar, vegetable fat, hydrolyzed vegetable protein, salt, yeast extract, white pepper, flavoring identical to natural of meat, citric acid, monosodium glutamate, disodium inosinate, disodium guanylate, silicon dioxide, tricalcium phosphate, and sodium citrate. | Meat flavor seasoning. |
